# Complete Degradation and Detoxification of Ciprofloxacin by a Micro-/Nanostructured Biogenic Mn Oxide Composite from a Highly Active Mn^2+^-Oxidizing *Pseudomonas* Strain

**DOI:** 10.3390/nano11071660

**Published:** 2021-06-24

**Authors:** Li Li, Jin Liu, Jie Zeng, Jiaoqing Li, Yongxuan Liu, Xiaowen Sun, Liangzheng Xu, Lin Li

**Affiliations:** 1 State Key Laboratory of Agricultural Microbiology, Huazhong Agricultural University, Wuhan 430070, China; lily999wy@126.com (L.L.); liujinlj1987@163.com (J.L.); zjzengjiezj@163.com (J.Z.); hzaulyx@webmail.hzau.edu.cn (Y.L.); sunxiaowen525@webmail.hzau.edu.cn (X.S.); 2 Guangdong Provincial Key Laboratory of Conservation and Precision Utilization of Characteristic Agricultural Resources in Mountainous Areas, Jiaying University, Meizhou 514015, China; lijiaoqing12@126.com

**Keywords:** biogenic manganese oxide, ciprofloxacin, degradation, antibiotic contamination

## Abstract

Ciprofloxacin (CIP), as a representative broad-spectrum antibiotic, poses a major threat to human health and the ecological environment as a result of its abuse and emissions. In this study, a highly active Mn^2+^-oxidizing bacterium, *Pseudomonas* sp. CCTCC M2014168, was induced to form micro-/nanostructured biogenic Mn oxide (BMO) aggregates through continuous culturing with 1 mmoL^−1^ Mn^2+^. Following the characterization of Mn^4+^ oxides and the micro-/nanostructures by scanning electron microscopy, high-resolution transmission electron microscopy and X-ray diffraction assays, the BMO composites were subjected to CIP degradation and detoxification in laboratory trials. High-performance liquid chromatograph (HPLC) analysis identified that the BMO composites were capable of completely degrading CIP, and HPLC with a mass spectrometer (LC/MS) assays identified three intermediates in the degradation pathway. The reaction temperature, pH and initial ciprofloxacin concentration substantially affected the degradation efficiency of CIP to a certain extent, and the metal ions Mg^2+^, Cu^2+^, Ni^2+^ and Co^2+^ exerted significant inhibitory effects on CIP degradation. A toxicity test of the degradation products showed that CIP was completely detoxified by degradation. Moreover, the prepared BMO composite exhibited a high capacity for repeated degradation and good performance in continuous degradation cycles, as well as a high capacity to degrade CIP in real natural water.

## 1. Introduction

Antibiotics have been globally used as important or even irreplaceable clinical drugs for curing infections or other diseases in humans and animals. Because of the large population, the use of various antibiotics is also tremendous in hospitals and stockbreeding fields in China, ranking at the forefront of the nation in terms of total amount of production and application [1,2]. However, the major problems associated with antibiotics are abuse and untreated discharge, which cause drug resistance and pose serious threats to the ecological environment and human health. Therefore, antibiotic pollution has become a serious public concern in China.

Ciprofloxacin (CIP) is a synthetic, third-generation quinolone antibiotic. As a representative broad-spectrum antibiotic, CIP exhibits antibacterial activity not only against a variety of Gram-positive bacteria (such as some pathogens of the genera *Staphylococcus*, *Pneumococcus* and *Streptococcus*) but also against certain Gram-negative bacteria, and it has inhibitory effects on some species of *Mycobacterium*, *Escherichia coli* and *Mycoplasma hominis* [2,3,4], leading to extensive use of this antibiotic with increasing frequency in clinical treatments. Unfortunately, due to its structural stability, CIP is unable to be completely metabolized by the human body, resulting in a number of residues that are released to the natural environment. Meanwhile, large quantities of unused or expired CIP drugs are conventionally discharged directly into the environment as wastes. As a result, CIPs or incompletely degraded metabolites normally diffuse rapidly in water bodies, or are absorbed by minerals and organic matter after spreading to the soil, and accumulate there in the form of hydrochloride, ultimately posing an ecotoxicological effect [5,6,7]. Additionally, the increase in antimicrobial resistance caused by the abuse of CIP also has a detrimental effect on the natural environment [8].

Manganese oxides are common minerals that are widely distributed in soil, seawater, neutral water and various sediments [9]. Mn oxide minerals are generally recognized to be formed by chemical and biological catalysis and oxidation in natural systems, and biogenic Mn^2+^ oxidation mediated by certain microorganisms dominates the biomineralization of Mn oxides [10,11]. Because of their high ionic valence state, Mn oxides (Mn^3+^ and Mn^4+^) have a high oxidative potential and are therefore potential oxidants for degrading certain natural and exogenous compounds [12]. A chemically synthetic Mn oxide (KMn_8_O_16_) has previously been used to oxidize CIP [13]. The ability of a biogenic Mn oxide (BMO) produced by an Mn^2+^-oxidizing bacterium to oxidize CIP has also been evaluated [14]. These Mn oxide-based treatments provide additional technical approaches for CIP degradation in principle. However, no effort has been undertaken yet to validate the degradability of CIP by a nanostructured BMO composite. Moreover, chemosynthetic Mn oxides should be considered to generally disregard the biodegraded activity of an Mn^2+^-oxidizing bacterium itself (i.e., *Pseudomonas* sp.) towards CIP, or facilitate regeneration capability of BMOs for continuous operations.

In this study, we investigated the degradation capability of CIP by a micro-/nanostructured BMO composite formed by a highly active Mn^2+^-oxidizing *Pseudomonas* strain. We prepared BMO aggregates through continuous culturing of the bacterial cells under prolonged low concentrations of Mn^2+^, characterized the as-prepared BMOs by X-ray diffraction (XRD) analysis, identified the micro-/nanostructured characteristics of the BMOs via high-resolution transmission electron microscopy (HRTEM) assays and further assayed their degradation capability on CIP in laboratory trials. Optimization experiments were performed to investigate the effects of temperature, pH, initial concentration of CIP and several metal ions on the degradation efficiency. *Staphylococcus aureus* and *Pseudomonas aeruginosa* were used as indicators of Gram-positive and Gram-negative bacteria, respectively, to bioassay the antibacterial activity of CIP-degraded products. Moreover, studies were also conducted to determine the degradation efficiency of CIP by the BMO composite under continuous and repeated performance, as well as the degradation of real natural lake water containing CIP. The possible metabolic pathway of the degradation was also discussed.

## 2. Materials and Methods

### 2.1. Reagents

All chemicals were of analytical grade and were used as received without further purification. CIP (purity ≥ 99%) was purchased from Sigma-Aldrich Co. LLC., Shanghai, China. Acetonitrile and methanol at the chromatographic level (purity ≥ 99.8%) were purchased from MREDA (Beijing, China). CIP was dissolved in an acidic aqueous solution and filtered through a 0.22-μm filter as a stock solution (5 mg·mL^−1^).

### 2.2. Growth and Manganese Oxidation Activity Determination

*Pseudomonas* sp. CCTCC (China Center for Type Culture Collections) M2014168 was used to prepare the BMO aggregates under laboratory trial. Cells were routinely cultured in Lept medium [15] at 28 °C. For determination of the Mn^2+^-oxidizing activity of cell cultures, the cells were incubated in 100 mL Lept broth, containing 1 mmol·L^−1^ Mn^2+^ at the final concentration, with shaking at 150 rpm to allow continuous 96 h of culture. The cell density at 600 nm (OD_600_) was measured at 8 h intervals using an ultraviolet/visible (UV/VIS) spectrophotometer (DU-800 Nucleic Acids/Protein Analyzer, Beckman Coulter, CA, USA). The Mn oxidation activity (refers to the Mn oxide concentration formed by bacterial cells) was determined according to a previously described method [16].

### 2.3. Preparation of BMO/Bacteria Composites

Overnight-cultured *Pseudomonas* sp. M2014168 cells were inoculated at 1% inoculum size (*v*/*v*) into 200 mL Lept broth supplemented with 1 mmoL^−1^ Mn^2+^ at the final concentration to allow continuous incubation with shaking at 150 rpm for 96 h at 28 °C. The suspension cultures were then harvested, and the BMO aggregate composites that were yielded were rinsed three times with double-distilled water (ddH_2_O), freeze-dried to obtain the composite powders, and stored at 4 °C until use. For CIP degradation, the as-prepared composites were dissolved in ddH_2_O to a final concentration of 1.28 mmol L^−1^ (Mn oxide concentration in composites) in the degradation reaction solutions unless stated otherwise.

### 2.4. Electron Microscope and Power X-ray Diffraction Assays

Scanning electron microscopy (SEM, Hitachi S-4700, Hitachi, Tokyo, Japan) observation of the BMO aggregates was performed following previous protocols [17]. HRTEM (JEM-2100F, JEOL, Tokyo, Japan), equipped with an energy-dispersive spectroscopy (EDS) detector, was used to investigate the microstructure of the BMO sample and analyze its elements. HRTEM was operated at an acceleration voltage of 200 kV, and sample preparation and HRTEM observation were performed by following the manufacturer’s manual. Bacterial BMOs were extracted and then freeze-dried to obtain Mn oxide powders for X-ray diffraction (XRD) as previously described [18].

### 2.5. Degradation of CIP

Degradation experiments on CIP were performed in 100 mL Erlenmeyer flasks on a laboratory scale under shaking at 150 rpm. Each equal amount of BMO composite solution (at a final concentration of 1.28 mmol·L^−1^) was allocated to a reaction solution containing 1 μg·mL^−1^ CIP. The degradation reaction time was routinely set to 12 h. After the degradation reaction, the supernatant of the reaction solutions was harvested by centrifugation, and an equal volume of ethyl acetate was added to obtain the organic phases, which were further subjected to rotary evaporation experiments to condense the solutions. The prepared samples were redissolved in acetonitrile and filtered through a 0.22-μm microporous membrane. CIP was separated and assayed using a high-performance liquid chromatograph (HPLC) (Shimadzu LC20-AT, Shimadzu, Kyoto, Japan) equipped with a C18 nonpolar reversed-phase bonded column (4.6 nm × 250 mm × 5 μm). The samples were eluted using an isocratic elution protocol, with parameters set at 15 min as the elution time. The flow rate was 1.0 mL min^−1^, and 279 nm was the detection wavelength, following previously described procedures [19]. The CIP degradation rate was calculated as follows [20]:

(1) $$\mathrm{Degradation}\;\mathrm{rate}\;=\frac{Ci-Cf}{Ci}\times 100\%$$

where *C_i_* denotes the initial CIP concentration (μg·mL^−1^) and *C_f_* denotes the final CIP concentration after degradation (μg·mL^−1^).

The content of CIP-degraded products was determined by HPLC coupled with a mass spectrometer (LC/MS) using a reversed-phase Waters ACQUITY BEH C-18 (Waters, Milford, MA, USA) column (2.1 mm × 50 mm × 1.7 μm) on an Agilent 1200/6460 LC/QQQ system (6460, Agilent, CA, USA). The mass detector included an ion source in positive ion mode, with the scanning mass-to-charge ratio ranging from 100 *m*/*z* to 500 *m*/*z*. The CIP degradation products were determined according to previously described procedures [21].

For optimization experiments on CIP degradation, temperatures ranging from 15 °C to 45 °C, pH values ranging from 4 to 7, initial CIP concentrations in the range of 1 to 20 μg·mL^−1^ and 1 mmoL^−1^ metal ions (Mg^2+^, Co^2+^, Ni^2+^, and Cu^2+^) were investigated for their effects on CIP degradation efficiency by the as-prepared BMO composites.

### 2.6. Determination of MIC of CIP

The antibacterial activity of CIP was assessed by the minimum inhibitory concentration (MIC), which was determined using the *Escherichia coli* K12 strain as the bacterial indicator, following a double-dilution protocol as described previously [22].

### 2.7. Inhibition Zone Test

In the inhibition zone experiments, *E. coli* K12 was also used as the bacterial indicator. An appropriate amount of *E. coli* K12 cells was evenly plated on freshly prepared LB plates, and then 4 sterile Oxford cups were equidistantly placed on the plates. The CIP-degraded solutions were filtered using a 0.22 μm microporous membrane under sterile conditions, and the same volume of filtrates was allocated to each cup to allow incubation at 37 °C for 24 h. The diameters of the inhibition zones were then measured.

### 2.8. Growth Inhibition Experiments

Strains *S. aureus* KCTC (Korean Collection for Type Cultures) 1621 and *P. aeruginosa* ATCC (American Type Culture Collections) 15,442 were used as Gram-positive and Gram-negative bacteria indicators, respectively, for the bioassays of growth inhibition by CIP-degraded products. The CIP degradation solutions after 12 h of reaction on 1 μg·mL^−1^ CIP, 1 μg·mL^−1^ CIP solution (positive control) and sterile ddH_2_O (negative control) were added at equal volumes to each 100 mL of freshly prepared LB broth, and then inoculated with the indicative bacterial cells at equal inoculation sizes. The cell suspensions were then incubated at 20 °C on a shaking incubator at 150 rpm for 72 h. The OD_600_ was measured at a time interval to monitor the growth of the cells.

### 2.9. Continuous and Repeated Degradation Performance

Continuous degradation experiments were performed in 100 mL Erlenmeyer flasks under laboratory trials. The degradation reactions were carried out at 45 °C, pH 4.0, 150 rpm and an initial CIP concentration of 1 μg·mL^−1^, with a total of 4 degradation cycles, and each cycle was run for 12 h. After each cycle degradation reaction, the BMO composites were harvested by centrifugation, washed 3 times with ddH_2_O and used directly for the next cycle of degradation. The degradation rate of each CIP-degradation cycle was determined and was calculated by Equation (1).

### 2.10. Degradation on CIP-Containing Natural Water

Real natural water was taken from an urban inner lake in Wuhan, China. Before the degradation experiments, the lake water was physically filtered to remove large particles. Prior to degradation, CIP (purity) was added to the water samples to a final concentration of 1 μg·mL^−1^, an appropriate amount of as-prepared BMO composite was added to the reaction solution, and the reactions were run for 7 days. The content of CIP in the reaction solutions was determined at each 24 h interval.

## 3. Results

### 3.1. Formation and Characterization of BMO Aggregate Composites

**Figure 1:**
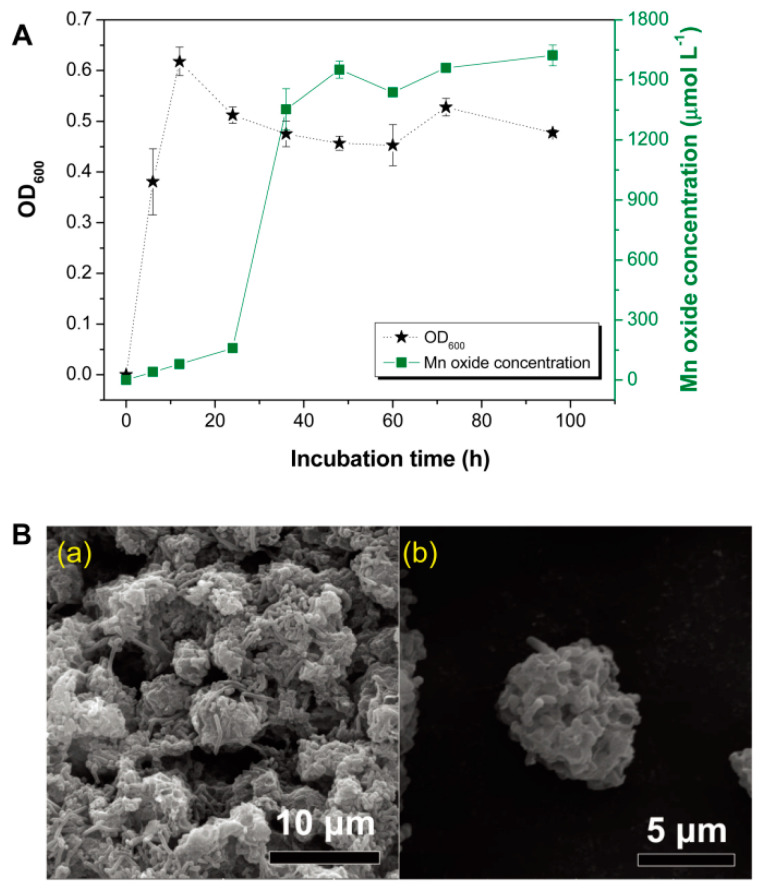
(**A**) Timecourse of Mn^2+^-oxidizing activity of the BMO cultures; (**B**) SEM micrograph of the formed aggregates in 48 h cultures (**a**); and SEM micrograph of a representative aggregate (**b**).

A soil-borne *Pseudomonas* strain, CCTCC M2014168, was previously identified in brown soil that surrounded Fe-Mn nodules [17]. Mn oxidation activity (refers to Mn oxide concentration) monitoring of this bacterium during the 96 h incubation timecourse showed that the activity was sharply increased in 24 h, then was maintained at high levels across the culturing timecourse (Figure 1A). Interestingly, this bacterium could form a kind of aggregate composite consisting of Mn oxides and bacterial cells. SEM observation showed that, after 48 h of culture, relatively regular micromorphological aggregates formed in the cell culture suspension and their surfaces were covered with biofilms (Figure 1B(a)). After culturing the cells for 96 h, the diameter of the aggregates that were formed remained approximately 4–10 µm, and the bacteria attached to and embedded in the aggregates were easily distinguished (Figure 1B(b)).

**Figure 2:**
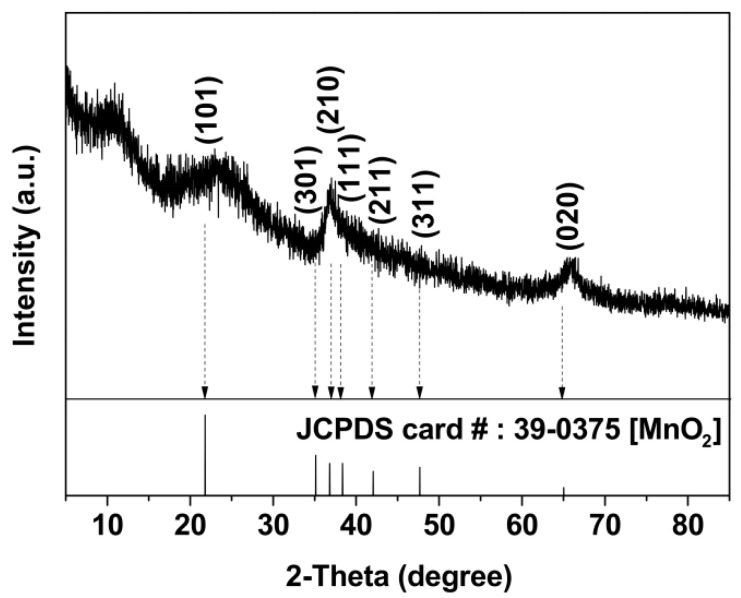
XRD pattern of Mn oxides of the BMO aggregates.

XRD assays were performed to verify the types of Mn oxides in the microspherical aggregates. The XRD pattern of the aggregate samples showed that the diffraction peaks were attributed to the (101), (301), (210), (111), (211), (311) and (020) planes corresponding to natural ramsdellite MnO_2_ (JCPDS card no. 39-0375) (Figure 2). Therefore, these results confirmed that the microspherical aggregates that were formed were composed mainly of ramsdellite (MnO_2_).

**Figure 3:**
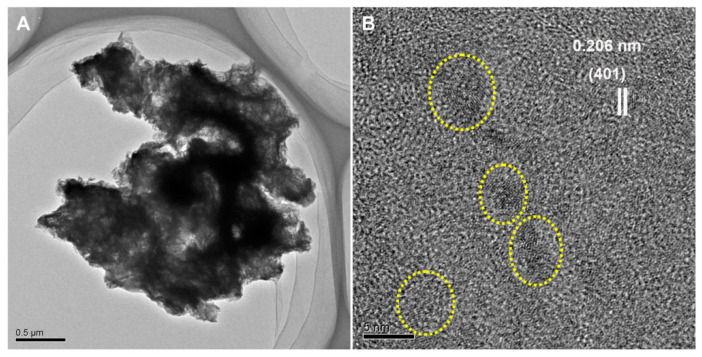
(**A**) Typical HRTEM images of a randomly selected BMO aggregate particle; (**B**) measured lattice spacings of the micro-/nanostructured BMO aggregate matter.

HRTEM was performed to investigate the fine microstructure of the formed BMO aggregates. Figure 3A shows that a randomly observed single aggregate particle had an irregular microspherical structure, with a particle diameter of approximately 4.5 μm; however, multiple groups of nanocrystalline particles with a diameter of 5 ± 1 nm were dispersed and embedded in the organic matter (Figure 3B), and the lattice fringe of 0.206 nm corresponded to the *d* value of the (401) plane-spacing in the ramsdellite-type MnO_2,_ suggesting that the BMO aggregates formed by bacterial mineralization were micro-/nanostructured-type composites.

### 3.2. Degradation of CIP by the BMO Composites and Constructive Degradation-Metabolic Pathway

**Figure 4:**
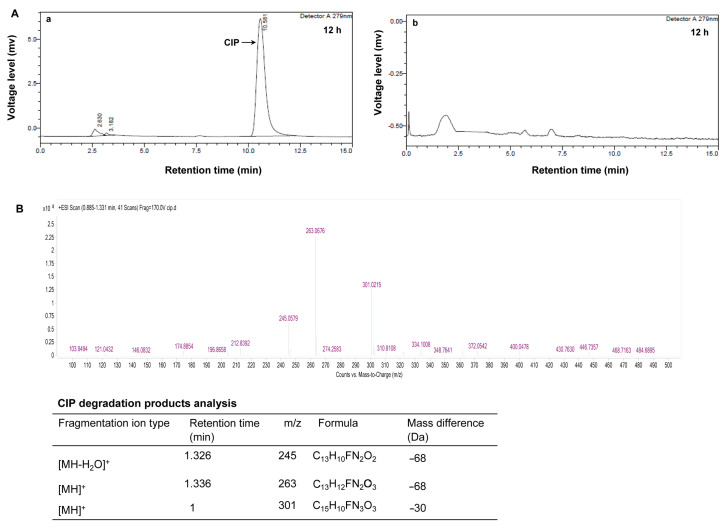
(**A**) HPLC chromatographs of CIP degraded by the BMO composites: (**a**) without the BMO composites; (**b**) with the BMO composites for 12 h. (**B**) LC/MS assays of the CIP-degraded intermediates.

To investigate the degradability of the as-prepared BMO composite on CIP, based on pre-experiments using a certain amount of the composites to treat CIP-containing solutions at a final concentration of 1 μg·mL^−1^, we selected BMO composites loaded at a final concentration of 1.28 mmol·L^−1^ for degradation experiments; HPLC assays were performed to measure the residual CIP after the degradation reactions. Compared to a clear specific CIP peak at a retention time of 10.5 min, detected in HPLC assays of the control supernatant fraction (without the BMO composites) after a 12 h reaction (Figure 4A(a), indicated by the arrow), no CIP specific peak was detected in the supernatants of the reaction solutions containing the BMO composites when the reaction was conducted at the same timecourse (Figure 4A(b)), suggesting that CIP was completely eliminated from the solvents. To clarify whether this removal was due to the degradation of the BMO composites (rather than biosorption) in terms of the appearance of decomposed intermediates, the supernatants at different degradation–reaction times ranging from 0.5–12 h were sampled and subjected to immediate HPLC assays. As a result, three intermediate product peaks at retention times of 1, 1.326 and 1.336 min were detected. We then performed LC/MS assays to identify these intermediates. As shown in Figure 4B, these three intermediates had *m/z* values of 245, 263 and 301, and their formulas were predicted by searching and aligning with the database and referring to a previous investigation [13]. In principle, these results not only indicate that the as-prepared BMO composites were capable of eliminating CIP completely but also suggest that the BMO composite removed CIP by degradation.

**Figure 5:**
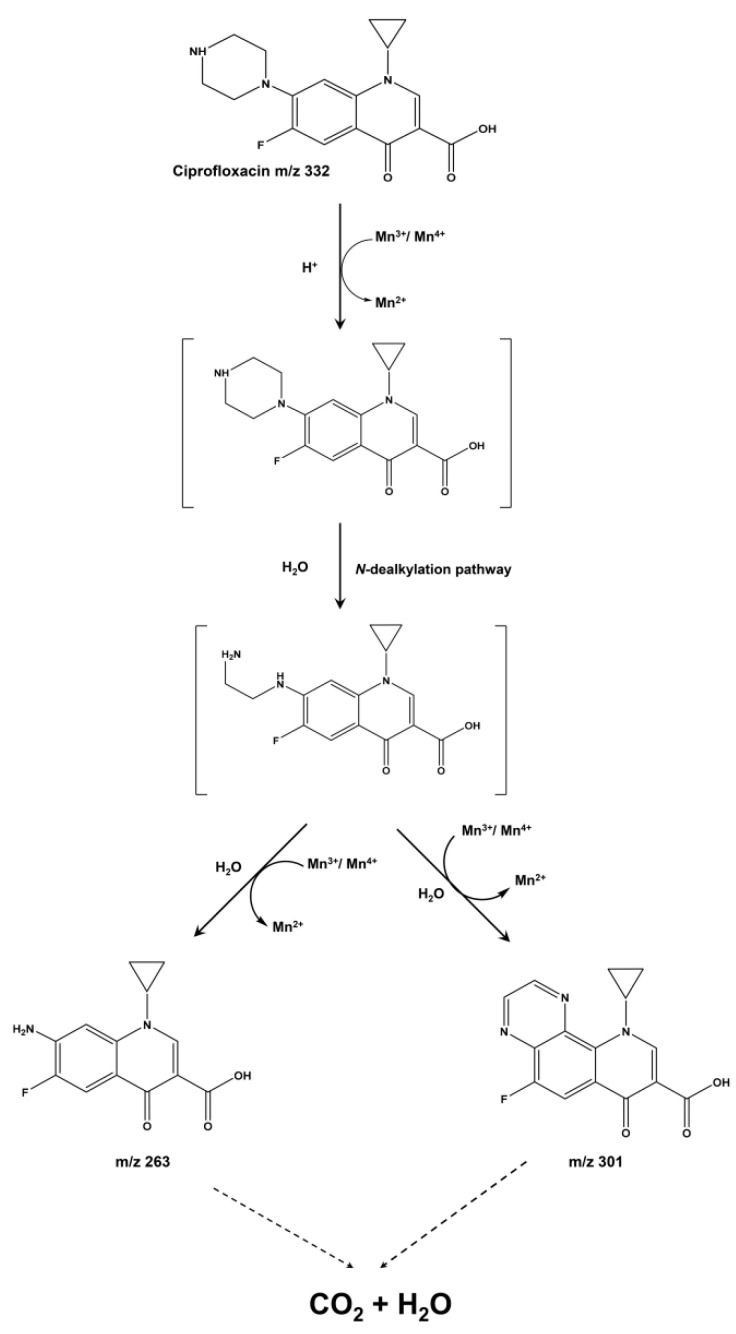
Constructive degradation-metabolic pathway of CIP.

Based on previous reports and the LC/MS assay patterns of CIP-degraded products, the metabolic pathway for the degradation of CIP by the BMO composites is constructed as follows (Figure 5). The molecular structure of CIP is composed of two parts: a quinolone structure containing a benzene ring and a piperazine ring structure containing a six-membered N ring [23], and the degradation of CIP by the BMO composite could be conducted mainly through destroying the piperazine ring structure [24]. There are two active sites, N_1_ and N_4_, on the piperazine ring. The BMO composites have a high oxidation potential due to the presence of high valence Mn (Mn^4+^), and they attack the N_1_ active site on the piperazine ring to require the transfer of two electrons, leading oxidation to form a double bond structure and undergo hydrolysis. Similarly, the N_4_ active site on the piperazine ring also loses electrons to form a double bond structure, and hydrolysis leads to ring-opening formation of imine ions, yielding an intermediate metabolite called dibenzyl CIP (*m*/*z* = 306). A common reaction is known as the dealkylation of CIP [25]; the ensuing degradation is diverse. One degradation path is to continue the oxidative hydrolysis of the molecules on the two active sites of the piperazine ring in the same way and finally generate a product with a mass-to-charge ratio of 263 (7-amino-1-cyclopropyl-6-fluoro-4-oxo-1,2,3,4-tetrahydroquinoline-3-carboxylic acid). The carboxyl group on the quinolone ring of this substance will continue to remove a molecule of H_2_O to produce an intermediate product with *m*/*z* of 245; the other route is to dehydrogenate and polymerize the two active centers on the piperazine ring of dimethyl ciprofloxacin (*m*/*z* = 306) to form double bonds under the action of the high oxidation potential of Mn oxides. A closed six-membered ring structure with C-C conjugated double bonds is formed (*m*/*z* = 301). Some previous investigations have suggested that the production of this substance may be related to the C atom at position eight on the quinolone [26,27].

### 3.3. Effect of Reaction Conditions on CIP Degradation

**Figure 6:**
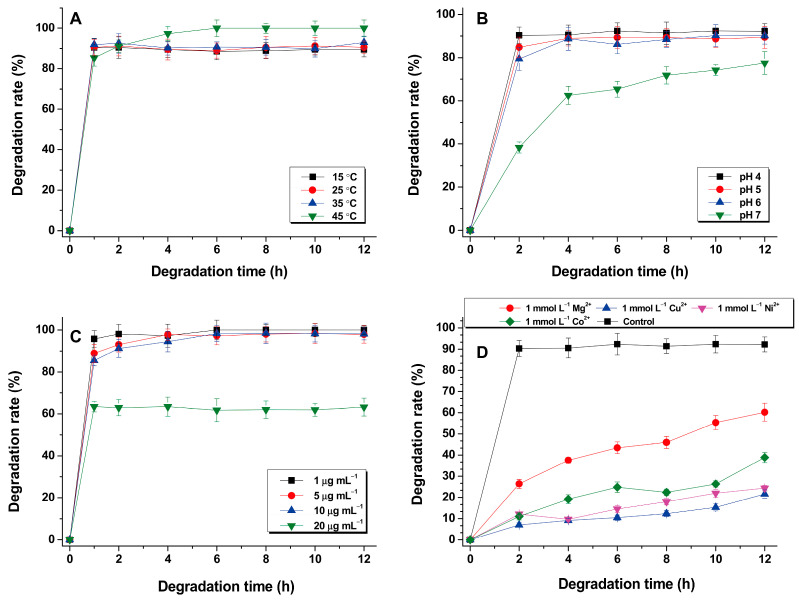
Effects of different temperatures (**A**), pH values (**B**), initial CIP concentrations (**C**) and metal ions (**D**) on CIP degradation by the BMO composites.

The effect of reaction temperature on the degradation efficiency of CIP by the BMO composites was evaluated from 15 °C to 45 °C. Figure 6A shows that CIP degradation was steady at all tested temperatures, with a slight increase in the degradation rate from approximately 90% to 100% following increases in temperature from 15 °C to 45 °C, reflecting that the degradation system can be steadily performed over a relatively wide moderate-temperature range. Figure 6B shows that CIP was degraded under all tested pH conditions, but a pH of 4.0 maximized the degradation rate of CIP at over 90% for a 12 h reaction. Increasing the pH from acidic to neutral tended to reduce the degradation efficacy.

Figure 6C shows that a loading of the BMO composites at the final concentration of 1.28 mmol·L^−1^ was able to completely degrade CIP at three initial concentrations of 1.0, 5.0 and 10.0 μg·mL^−1^; however, when the initial CIP was increased to 20.0 μg·mL^−1^, the CIP degradation rate declined to approximately 60%, remaining steady, although the reaction time increased from 1 h to 12 h.

Several common metal ions (Mg^2+^, Cu^2+^, Ni^2+^ and Co^2+)^ were selected to evaluate their effects on the degradation efficiency of CIP. As shown in Figure 6D, adding each metal ion alone at a final concentration of 1 mmol·L^−1^ significantly decreased the CIP degradation rate, with an order of inhibitory ability of Cu^2+^ > Ni^2+^ > Co^2+^ > Mg^2+^. Our current hypothesis to explain this result is that it may be due to the adsorption of metal ions, resulting in the surface of the active site being occupied by metal ions, thereby affecting CIP degradation activity [28].

### 3.4. Antibacterial Activity Evaluation of CIP Degradation Products

The minimum inhibitory concentration (MIC) refers to the lowest effective concentration of antibiotics to inhibit the growth of pathogenic microorganisms in vitro. MIC has been recognized as one of the important indicators for antibiotics to inhibit pathogenic bacteria [29]. The MIC of CIP determined against *E. coli* was located between 0.125 and 0.25 μg·mL^−1^ (Figure S1), which was consistent with a previous investigation [30].

Figure S2 shows the determination of the CIP-degraded product in the growth inhibition zone of the indicative *E. coli* K12 cells. The results show that the same volume of solutions containing the degraded CIP products at dilutions of 0, 1 and 2 times (*v*/*v* by sterile ddH_2_O) did not cause a visible inhibition zone (cups 1, 2, and 3, respectively; see Figure S2), whereas an inhibition zone 25 mm in diameter was clearly visible in the control (cup 4, Figure S2).

**Figure 7:**
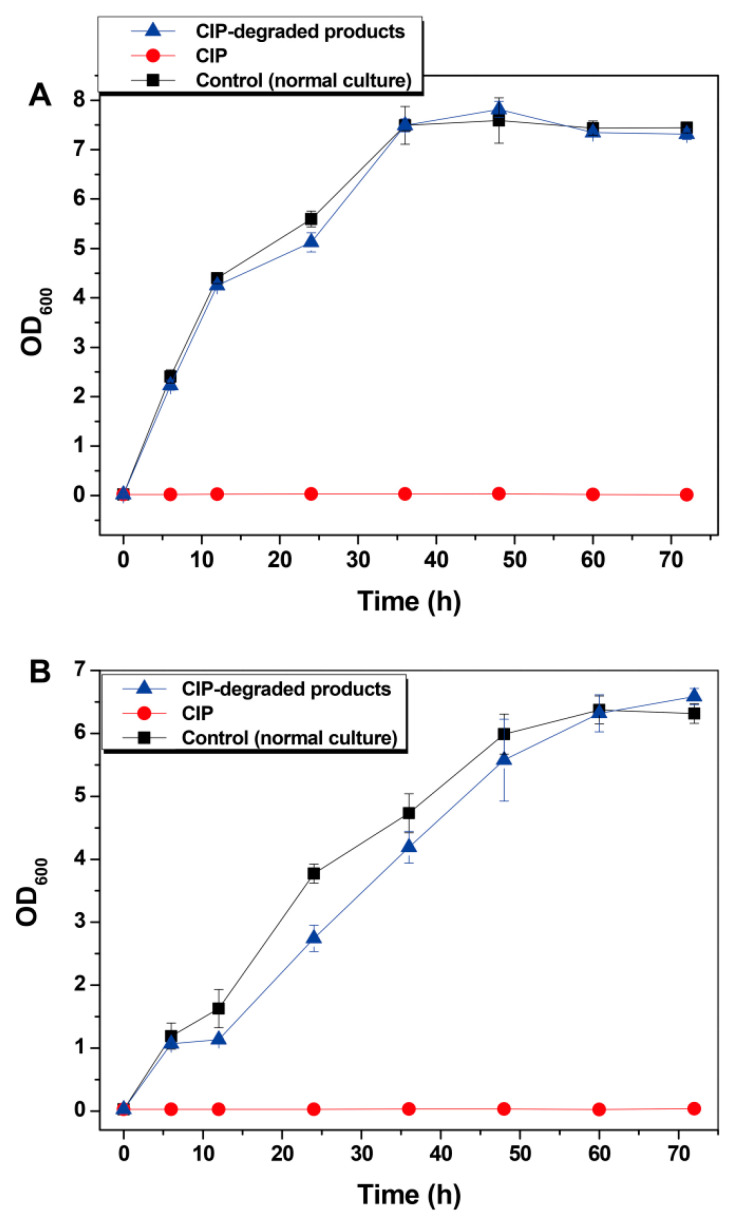
Growth effect of CIP-degraded products on *S. aureus* KCTC 1621 (**A**) and *P. aeruginosa* ATCC 15442 (**B**) over a 72 h culture timecourse.

*S. aureus* KCTC 1621 and *P. aeruginosa* ATCC 15442 were used as the indicative Gram-positive and Gram-negative bacteria, respectively, for the evaluation of growth inhibition by the CIP degradation products in a 72 h culture timecourse. Figure 7A shows that, compared with complete growth inhibition by CIP, the degradation products of CIP by the BMO composites did not cause an inhibitory effect on the growth of *S. aureus* KCTC 1621 cells, which exhibited a steadily increased growth pattern along with the growth pattern of the normal cell cultures (Control). Figure 7B shows similar profiles for the CIP-degraded products, the normal culture (Control) and CIP towards *P. aeruginosa* ATCC 15442 cells. These results indicate that CIP degradation by the BMO composites completely detoxified the degradation products in terms of antibacterial activity.

### 3.5. Continuous/Repeated Degradation

**Figure 8:**
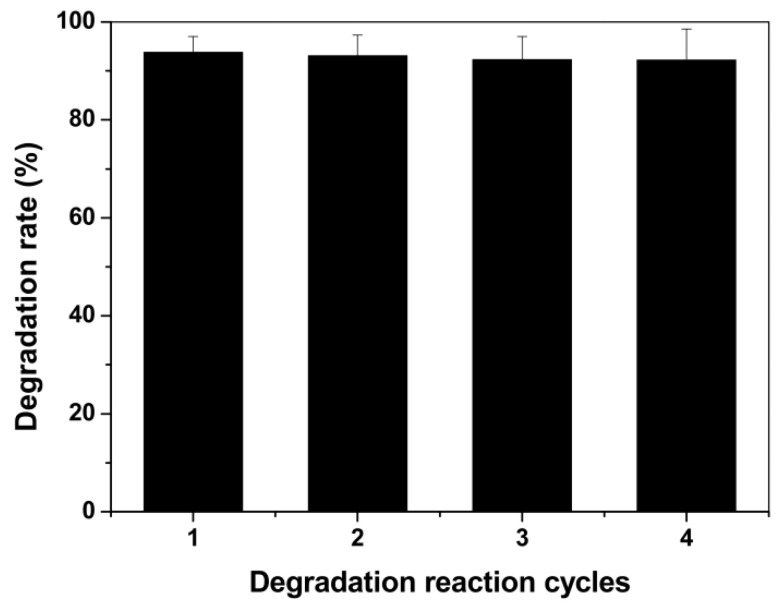
Continuous four-round degradation of CIP by the BMO composites.

Four rounds of continuously repeated degradation of CIP were performed to assess the CIP degradation efficacy in laboratory shake flasks. Figure 8 shows that the BMO composites maintained high CIP degradation activity in all four rounds, with degradation rates of 93.8%, 93.1%, 92.7% and 92.5% in rounds 1, 2, 3 and 4, respectively. These data demonstrate the serially reusable performance of the BMO composites in degrading CIP.

### 3.6. CIP Degradation on CIP-Containing Natural Water

**Figure 9:**
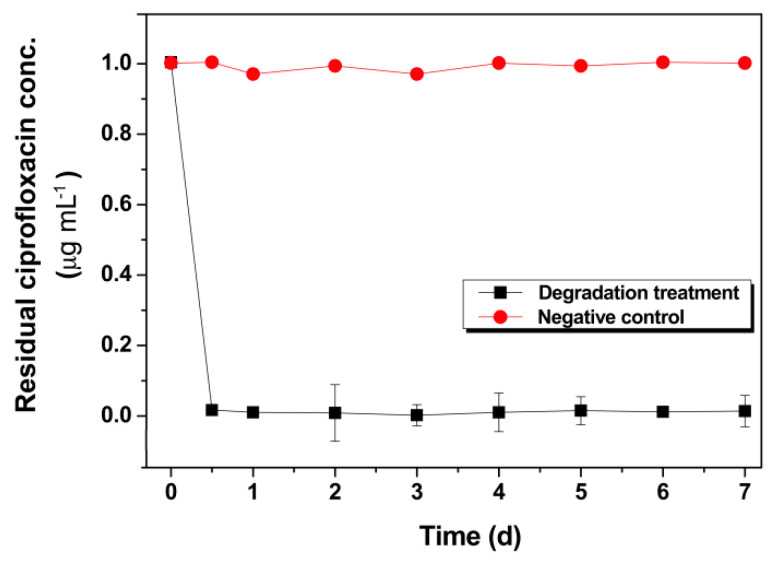
Degradation timecourse of CIP in natural lake water by the BMO composites.

Natural water bodies usually contain different kinds of impurity components to varying degrees, and the pH of the natural water body is generally higher than the optimum pH range for CIP degradation (pH 4 to pH 6). These factors could restrain the effectiveness of CIP degradation in real natural water. To investigate the degradability of the as-prepared BMO composite on CIP-containing natural water, we used urban lake water (pH 6.5) as the background liquid to evaluate the degradation efficacy of CIP in natural water in laboratory shake-flask trials. CIP was added to a final concentration of 1 μg·mL^−1^ in natural water, and the degradation reactions were run for 7 continuous days. HPLC assays were performed to determine the residual CIP content of the supernatants from the initial 12 h and the reactive solutions of each day. Figure 9 shows that CIPs were degraded to a detectable low-limit level from the initial 12 h and then maintained across the treatment timecourse. In contrast, CIP was steady at an initial concentration in the control. These results suggest the potential of the developed BMO composites to treat real natural waters containing CIP.

## 4. Conclusions

A micro-/nanostructured BMO composite was prepared from a highly active Mn^2+^-oxidizing *Pseudomonas* bacterium. After characterization of the Mn oxide pattern and the morphological microstructure of the formed microspherical aggregates by SEM, HRSEM and XRD assays, the BMO composites were demonstrated to be able to completely degrade CIP in various laboratory shake-flask trials. Degradation can be performed over a wide range of pH values (pH 4–6) and temperatures (15–45 °C), with an initial CIP concentration of 10 μg·mL^−1^ when 1.28 mmol L^−1^ BMO composites are loaded. HPLC and LC/MS assays demonstrated the elimination of CIP due to complete CIP degradation without the accumulation of intermediate molecules. Residual antibacterial bioassays on several indicative bacterial strains verified the complete elimination of toxicity from the degradation products. Moreover, the composite that was developed also exhibited good continuous performance and a good capacity to detoxify real natural waters containing CIP, suggesting potential for further investigation in large-scale or continuous degradation processes.

## Data Availability

Data available in a publicly accessible repository.

## Supplementary Materials

The following are available online at https://www.mdpi.com/article/10.3390/nano11071660/s1, Figure S1. Determination of CIP MIC on *Escherichia coli* cells using gradient double diluted CIP solutions. Tube 1–9, CIP at concentrations of 1–0.00390625 µg mL^−1^; Tube 10, no CIP; Tube 11, no *E. coli* cells. Figure S2. Inhibition zone experiment of CIP degradation products on Escherichia coli cells. Cup 1, original CIP degradation solution; Cup 2, double-diluted CIP degradation solution; Cup 3, triple-diluted CIP degradation solution; Cup 4, control solution (1 µg mL^−1^ CIP).
