# Supplementary material for: Complete Degradation and Detoxification of Ciprofloxacin by a Micro-/Nanostructured Biogenic Mn Oxide Composite from a Highly Active Mn2+-Oxidizing Pseudomonas Strain
Source: Nanomaterials (Basel). 2021 Jun 24;11(7):1660. doi: 10.3390/nano11071660 (PMC8304510; doi:10.3390/nano11071660)
Supplement: Supplementary file 1 [file nanomaterials-11-01660-s001.zip › nanomaterials-1255234-supplementary.pdf]

Supplement Materials

Complete Degradation and Detoxification of Ciprofloxacin by a Micro-/Nanostructured Biogenic Mn Oxide Composite from a Highly Active Mn^{2+}-Oxidizing *Pseudomonas* Strain

Li Li, Jin Liu , Jie Zeng, Jiaoqing Li, Yongxuan Liu, Xiaowen Sun, Liangzheng Xu and Lin Li

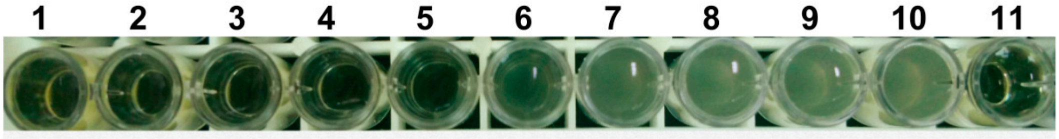

Figure S1. Determination of CIP MIC on *Escherichia coli* cells using gradient double diluted CIP solutions. Tube 1–9, CIP at concentrations of 1–0.00390625 μg mL^{-1}; Tube 10, no CIP; Tube 11, no *E. coli* cells.

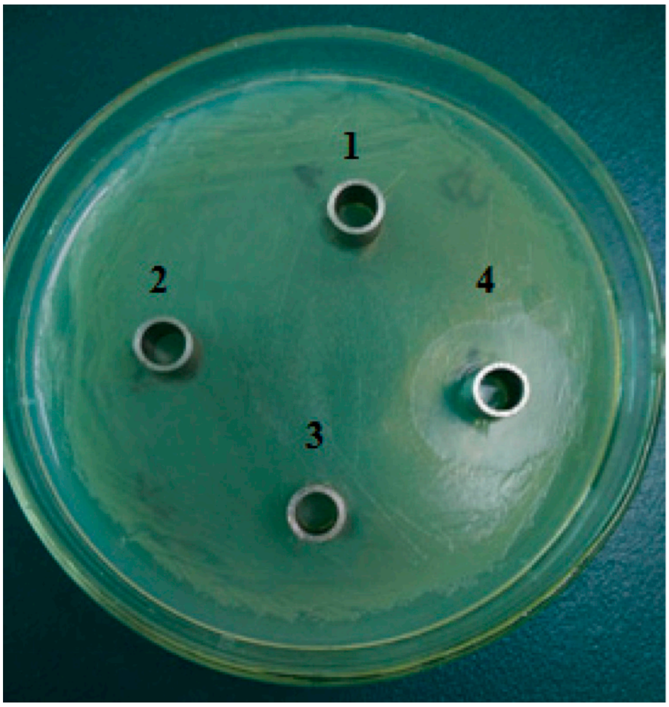

Figure S2. Inhibition zone experiment of CIP degradation products on *Escherichia coli* cells. Cup 1, original CIP degradation solution; Cup 2, double-diluted CIP degradation solution; Cup 3, triple-diluted CIP degradation solution; Cup 4, control solution (1 μg mL^{-1} CIP).
